# Detection of QTLs regulating the second internode length in rice dwarf mutant *d1*

**DOI:** 10.1270/jsbbs.24036

**Published:** 2024-12-03

**Authors:** Quynh T. Ha, Sandar Moe, Vincent Pamugas Reyes, Kazuyuki Doi, Kotaro Miura, Mio Mizushima, Akiteru Maeno, Katsutoshi Tsuda, Keisuke Nagai, Motoyuki Ashikari

**Affiliations:** 1 Department of Plant Production Sciences, Graduate School of Bioagricultural Sciences, Nagoya University, Furocho, Chikusa, Nagoya, Aichi 464-8601, Japan; 2 Faculty of Bioscience and Biotechnology, Fukui Prefectural University, 4-1-1 Kenjojima, Matsuoka, Eiheiji-cho, Yoshida-gun, Fukui 910-1195, Japan; 3 Department of Gene Function and Phenomics, National Institute of Genetics, Mishima, Shizuoka 411-8540, Japan; 4 Department of Genetics, School of Life Science, Graduate University for Advanced Studies, Mishima, Shizuoka 411-8540, Japan; 5 Bioscience and Biotechnology Center, Nagoya University, Furocho, Chikusa, Nagoya, Aichi 464-8601, Japan

**Keywords:** QTL, internode, rice

## Abstract

Stem length is a crucial agronomic trait in rice breeding. The short stature of rice dwarf mutants is caused by shortening of internodes, resulting in five distinct internode elongation patterns: dn, dm, d6, nl and sh. Several genetic studies have been conducted; however, the genetic mechanisms underlying these internode elongation patterns remain unclear. In this study, we examined two Daikoku dwarf (*d1*) mutants, T65(*d1-1*) and Kin(*d1-7*), which display contrasting internode elongation phenotypes. Anatomical observation revealed that T65(*d1-1*) exhibits a dm-type internode elongation pattern due to the lack of the second internode counted from the top, while Kin(*d1-7*) shows a dn-type pattern with a relatively elongated second internode. To identify the genetic factors influencing these phenotypes, we conducted a quantitative trait locus (QTL) analysis using two F_2_ populations derived from reciprocal crosses between them. The QTL analysis showed that the second internode length is regulated by three QTLs on chromosomes 4, 5, and 6. Epistatic effects were observed through the analysis of F_3_ progenies, indicating that the combination of Kin(*d1-7*) alleles at these QTLs is associated with an increased second internode length. Furthermore, specific combinations of alleles result in varying degrees of elongation in the second internode, significantly impacting the internode elongation pattern. These findings contribute to a deeper understanding of the genetic factors influencing the internode elongation patterns in rice.

## Introduction

**Fig. 1.**
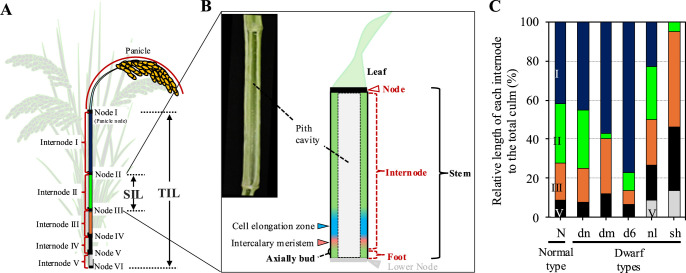
Rice stem and internode elongation patterns. (A) Illustration of a rice tiller at maturity. Numbering of internodes (left) and nodes (right) proceeds from the top to the base of rice culm. SIL, second internode length. TIL, total internode length. (B) Illustration of a rice phytomer, which is composed of a leaf, a stem and an axillary bud. The stem part includes domains of node, internode and foot, with only internode domain elongating by cell propagation in intercalary meristem and subsequent cell elongation in cell elongation zone. Illustration was modified from Nagai and Ashikari (2023). (C) Classification of internode elongation patterns in rice (re-drawn from Takeda 1977). Internode I (ocean blue). Internode II (green). Internode III (orange). Internode IV (black). Internode V (gray).

Stem length is an important agronomic trait in crop breeding, as dwarf stature not only enhances lodging resistance and harvest index but also improves the response to nitrogen fertilizers (Hedden 2003). The rice stem comprises nodes and internodes, with internode segments elongating due to cell propagation in the intercalary meristem and subsequent cell elongation in the cell elongation zone. Typically, modern rice cultivars possess 13–18 nodes on the main stem (culm) below the panicle, but only the upper four or five internodes elongate during the reproductive growth stage (Hoshikawa 1989, Yamaji and Ma 2014). Therefore, the rice stem length is primarily determined by the total length of these four or five internodes (Fig. 1A, 1B).

Currently, many rice dwarf and semi-dwarf mutants have been identified (Oryza base <https://shigen.nig.ac.jp/rice/oryzabase/>). The dwarf phenotype of these mutants typically arises from internode shortening. However, the degree of shortening in each internode is specific to each mutant, resulting in distinct internode elongation patterns among them. Takeda (1977) categorized several rice strains into a normal (N) or five dwarf types (dn, dm, d6, nl and sh) based on their upper four or five internode elongation patterns (Fig. 1C). Generally, numbering of internodes at maturing stage in rice proceeds from the top to the base of rice stem as Internode I–V (Hoshikawa 1989) (Fig. 1A, 1C). In the N-type, which is a typical internode elongation pattern of normal rice strains, the Internode I is the longest, with subsequent internodes gradually shortening from the first to the fifth (Fig. 1C). The total internode length of the dwarf types dn, dm, d6, nl and sh are generally shorter than that of the N-type, but they exhibit unique internode elongation patterns. The dn-type resembles the N-type in internode elongation pattern, with internodes reduced in proportion to those of the N-type. The dm-type is characterized by a significantly shortened Internode II. The d6 type also shows shortened internodes except for the Internode I, which is much longer than half of the stem length. The nl-type features a reduced Internode I and increased lower internodes. The sh-type lacks elongation in the Internode I, with the panicle enveloped in the leaf sheath. Despite these various internode elongation patterns reported in rice, the underlying mechanisms remain poorly understood. Thus, elucidating the genetic basis of these specific phenotypes not only enhances our understanding of internode elongation patterns but also contributes to rice breeding.

The dm-type internode elongation pattern, characterized by a specific reduction in the Internode II, is observed in many rice dwarf mutants such as *d1*, *d2*, *d11* and *d61* (Fujisawa *et al.* 1999, Hong *et al.* 2003, Tanabe *et al.* 2005, Yamamuro *et al.* 2000). Previous studies showed that the suppression of the Internode II elongation in the dm phenotype is regulated by various factors associated with multiple hormonal signaling pathways. This includes the brassinosteriod (BR) signaling pathway (Hong *et al.* 2003, Niu *et al.* 2022, Oki *et al.* 2009b, Tanabe *et al.* 2005, Yamamuro *et al.* 2000) and the gibberellin (GA) signaling pathway (Ashikari *et al.* 1999, Sakamoto *et al.* 2004, Ueguchi-Tanaka *et al.* 2000). To further understand the genetic basis of this internode elongation pattern, we utilized two Daikoku dwarf mutants (*d1*), namely T65(*d1-1*) and Kin(*d1-7*), in this study. We observed that T65(*d1-1*) plant exhibits a dm-type internode elongation pattern, whereas Kin(*d1-7*) plant show a dn-type pattern. An anatomical analysis suggested that T65(*d1-1*) plant might lack the Internode II. To identify genetic regions linked with the Internode II elongation, a quantitative trait locus (QTL) analysis using F_2_ populations derived from reciprocal crosses of T65(*d1-1*)/Kin(*d1-7*) and Kin(*d1-7*)/T65(*d1-1*) was conducted. As a result, we detected three QTLs on chromosomes 4, 5 and 6 associated with the elongation of the Internode II. To test the interaction among these QTLs, an analysis of F_3_ progenies was carried out. This revealed an epistatic effect, wherein specific combinations of alleles at three QTLs resulted in different elongation degree in the Internode II. Based on these findings, we have discussed about effects of the mutations and genetic backgrounds on the internode elongation patterns of the rice mutants.

## Materials and Methods

### Plant materials and growth conditions

Two *d1* mutant lines namely *d1-1* and *d1-7* were used in this study. The mutant line *d1-1* originated from the *Oryza sativa* subsp. *japonica* ‘Taichung65’ (T65) cultivar, and the mutant line *d1-7* came from the *Oryza sativa* subsp. *japonica* ‘Kinmaze’ (Kin) cultivar (Oki *et al.* 2009a). To specify the background cultivars of these mutants, we renamed *d1-1* as T65(*d1-1*) and *d1-7* as Kin(*d1-7*). T65(*d1-1*) belongs to the dm-type internode elongation pattern, whereas Kin(*d1-7*) belongs to the dn-type pattern (Fujisawa *et al.* 1999, Oki *et al.* 2009a). These mutant lines served as parental lines for reciprocal crosses, yielding F_1_ seeds, which were then self-pollinated to produce the F_2_ generation. In this study, two F_2_ populations, designated as T65(*d1-1*)/Kin(*d1-7*) F_2_ and Kin(*d1-7*)/T65(*d1-1*) F_2_ by its reciprocal crosses, were utilized for the QTL analysis. Subsequently, 11 F_3_ populations originated from F_2_ individuals carrying different genotype combinations were used for evaluation of QTL effect. In addition, the *d1* mutant lines, along with the original cultivars T65 and Kin were employed for the detailed phenotypic characterization and the anatomical observation.

Seeds from F_2_–F_3_ populations, along with *d1* mutant lines and original cultivars, were sterilized at 60°C for 10 minutes, pre-germinated at 30°C for three days, and then sown in perforated cell trays with cells measuring 1 cm in diameter and 2 cm deep. The seedlings were cultivated in a Nagoya University greenhouse and transplanted at one month old into the rice field at Togo, Nagoya University, Aichi, Japan (35°06ʹ36.5ʺN 137°05ʹ06.3ʺE). From June to October between 2021 and 2023, they were arranged in rows with eight plants each, spaced 20 cm apart, with 30 cm between rows. Standard agronomic practices for fertilization, pest, and disease control were followed to minimize yield loss.

### Numbering internodes and nodes at maturing stage

In this paper, we adopted the numbering scheme of internodes of Hoshikawa (1989), starting with Internode I at the top and continuing with Internodes II to V towards the base of the rice stem at the maturing stage (Fig. 1A). The numbering scheme of nodes was based on the order of phytomer units, which arrange from upper to lower parts in order: leaf, node, internode and axillary bud (Kawata *et al.* 1963). Consequently, the uppermost node below the panicle is Node I, which directly connects to Internode I. The subsequent nodes below the Node I are numbered as Node II–VI (Fig. 1A).

### Phenotypic characterization

At maturity, the plants were digged up, the remaining soil was cleaned from the roots, and the three tallest tillers from each plant were selected for phenotyping. To compare the internode elongation patterns of the *d1* mutant lines with the original cultivars, the length of each internode was measured as the distance between two successive nodes, collectively termed total internode length (TIL) (Fig. 1A). The relative length of each internode was determined by calculating its proportion of the TIL, using the formula: (length of each internode/TIL) × 100%. This calculation helps illustrate the internode elongation patterns. Additionally, panicle characteristics such as length and seed size were assessed; panicle length was defined as the distance from Node I to the tip of the panicle, and seed dimensions were measured with a Vernier caliper.

Given the contrasting elongation patterns of Internode II observed in T65(*d1-1*) and Kin(*d1-7*), we focused on it as a main quantitative parameter for QTL analysis. In this study, we refer to the length of Internode II as the second internode length (SIL) (Fig. 1A). To further understand the significant reduction in Internode II compared to other internodes in T65(*d1-1*), we also analyzed QTL to identify genomic regions linked to the length of Internode I, III, IV and V (Fig. 1A).

### Genotyping-by-sequencing (GBS)

The leaf samples were collected from the individual F_2_ and F_3_ plants after phenotyping. Genomic DNA from individual leaf sample was extracted using Dellaporta method (Dellaporta *et al.* 1983). The integrity of the DNA was checked via gel electrophoresis (with 0.8% agarose gel in 1X TAE buffer). The concentration of the double-stranded DNA was measured by QuantiFluor dsDNA System and Quantus Fluorometer (Promega, Madison, WI, USA), and adjusted into 20 ng/μL.

To construct the GBS sequencing library, the protocols of Reyes *et al.* (2021) and Furuta *et al.* (2017) were followed. In summary, 200 ng of genomic DNA from the individual samples were double-digested with *KpnI*–*MspI* enzymes (New England Biolabs Inc., Ipswich, MA, USA). After the double digestion step, unique barcoded adapters were ligated into the individual samples and were pooled into a single tube and purified using a QIAquick PCR Purification kit (Qiangen Sciences, Germantown, MD, USA). A modified flow cell primers containing designated index were used to amplify the multiplexed ligation products. The sequencing of the library was carried out using Illumina HiSeq (Illumina, Inc., San Diego, CA, USA).

To analyze the GBS sequence data, the raw sequences were preprocessed using cutadapt. The parameters that were used were: (i) removal of adapter sequences (“AGATCGGAAGAGCGG”) and (ii) a minimum read length criterion of 40 bases. The preprocessed sequences were then processed using the TASSEL-GBS pipeline 5.0, with the parameters of minimum locus coverage higher than 0.8. Reads were aligned to the IRGSP V1.0 *O. sativa* Nipponbare reference genome using BWA (Kawahara *et al.* 2013, Li and Durbin 2009). The reads that were obtained after the initial processing were further filtered to retain single-nucleotide polymorphisms (SNPs) that are polymorphic between parental lines.

### QTL analysis

A total of 143 F_2_ plants derived from the cross T65(*d1-1*)/Kin(*d1-7*) and 166 F_2_ plants derived from the cross Kin(*d1-7*)/T65(*d1-1*) were used for QTL analysis with 309 SNP markers. QTL analysis was based on interval mapping with “hk” option of “scanone” function implemented in R/qtl (Broman *et al.* 2003) and genetic distance between the adjacent markers was estimated by Kosambi mapping function (Kosambi 1943). A logarithm of odds (LOD) value of 3 was set as the threshold score, established using 1000 permutations to determine an empirical p-value of 0.05 (Churchill and Doerge 1994). QTL interaction was estimated by using “scantwo” function in R/qtl for two-QTL genome scan with the SIL data (Broman *et al.* 2003).

### Anatomical observation by micro-computed tomography (micro-CT)

Mature stem samples of the original cultivars and *d1* mutant lines were dissected from above Node II to beneath Node III (Supplemental Fig. 1A) at Nagoya University. The dissected samples were immediately put in tubes filling with fixation solution FAA (formalin : acetic acid : 50% ethanol = 5 : 5 : 90), then subjected to a vacuum at –0.08 MPa for 2 hours to ensure complete absorption of fixation solution. Subsequently, the sample tubes were delivered to National Institute of Genetics (NIG) for processing and micro-CT scanning.

The micro-CT scanning was modified from the protocols of Tsuda *et al.* (2017), and Maeno and Tsuda (2018) to suit the characteristics of mature rice stem samples. In summary, the stem samples were soaked in the fixation solution FAA for 1 week, then the fixation solution was replaced by 70% ethanol and stored at 4°C until observation. Before scanning, the samples were stained with the contrast agent with 1.0% [w/v] phosphotungstic acid in 70% ethanol for one month (compared to 1 week in the standard protocol). Subsequently, the samples were scanned using X-ray micro-CT imaging system at a tube voltage peak of 85 kVp and a tube current of 90 μA. The samples were rotated 360°C in steps of 0.24°, generating 1500 projection images of 992 × 992 pixels. The micro-CT data were constructed at an isotropic resolution of 8.0–8.5 μm. Three-dimensional tomographic images were obtained using OsiriX sorfware.

### Sequence and expression level of *D1* gene

To confirm sequence and determine endogenous expression level of *D1* gene, whole shoots from 5-leaf stage seedlings of the original cultivars and *d1* mutant lines were sampled and frozen in liquid nitrogen for RNA extraction. Total RNA was extracted using a Maxwell RSC Instrument (Promega), and first-strand cDNA was synthesized with the Omniscript RT Kit (Qiagen) and oligo(dT) 20 primers. For sequence confirmation, cDNA fragments of the *D1* (*Os05g0333200*) from the original cultivars and *d1* mutant lines were amplified by PCR using KOD Fx Neo (Toyobo). The amplified cDNA fragments from the *d1* mutant lines were introduced into plasmid vector pCR™-Blunt II-TOPO (Invitrogen) and transformed into *E. coli* XL10-Gold. Plasmids were isolated from *E. coli* cultures using the alkaline lysis method (Birnboim and Doly 1979), and the inserted cDNA regions were sequenced using capillary sequencing (ABI3730, Applied Biosystems). The sequence data were aligned using the Genetyx software and CLUSTAL-W program. To determine the expression level of the *D1* gene, quantitative PCR (qPCR) was performed using the StepOne Real-Time PCR System (Applied Biosystems) with Thunderbird Next SYBR qPCR Mix (Toyobo). Expression levels were normalized to that of RICE UBIQUITIN 2 (ubi-chr2). The cDNA of T65 sample was used as a template to generate standard calibration curves. The primers used for PCR amplification, cloning, sequencing and qPCR are listed in Supplemental Table 1.

### Statistical analysis

Statistical differences were analyzed by student’s t-test, or multiple-way ANOVA followed by Turkey’s multiple-comparison test using Prism 7 software.

## Results

### Characterization of the second internode (Internode II) of the *d1* mutant lines

**Fig. 2.**
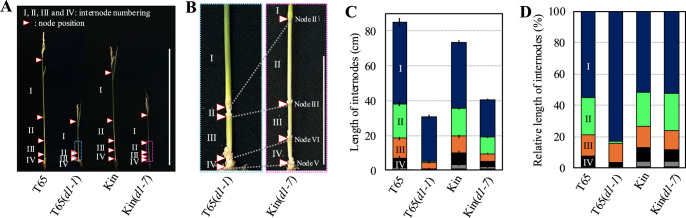
Internode elongation patterns of the original cultivars and *d1* mutant lines. (A) Internode morphology. The *d1* mutant lines show shorter internodes compared to the original cultivars. Bar, 1 m. (B) The magnified photos of the framed areas in (A). T65(*d1-1*) exhibits a significantly shortened Internode II, whereas Kin(*d1-7*) displays a relatively elongated Internode II. Bar, 10 cm. (C) Length of internodes. Values are means with SD, n = 6 in 2023. (D) Relative length of internodes to the total internode length (TIL), calculated from the quantitative data in (C). T65(*d1-1*) shows a dm-type internode elongation pattern, whereas Kin(*d1-7*) shows a dn-type pattern. T65, cultivar ‘Taichung65’. T65(*d1-1*), *d1* mutant line in background of T65. Kin, cultivar ‘Kinmaze’. Kin(*d1-7*), *d1* mutant line in background of ‘Kinmaze’.

We conducted a three-year survey (2021–2023) of the internode lengths in the *d1* mutant lines T65(*d1-1*) and Kin(*d1-7*) (Supplemental Table 2, Fig. 2). The internode phenotype trend between the two mutants remained stable (Supplemental Table 2). Specifically, the Internode I of T65(*d1-1*) was longer than that of Kin(*d1-7*), but the lower internodes of T65(*d1-1*) were significantly shorter than those of Kin(*d1-7*) (Supplemental Table 2, Fig. 2A, 2C). Notably, the Internode II of T65(*d1-1*) was significantly stunted, with the length less than 1cm (Supplemental Table 2, Fig. 2B, 2C), indicating a typical dm-type internode elongation pattern for T65(*d1-1*) (Figs. 1C, 2D). Conversely, the internode elongation pattern of Kin(*d1-7*) resembled that of the original cultivars T65 and Kin. The Internode I was the longest, and each successive internode became shorter from the top down to the base. This pattern suggests that Kin(*d1-7*) follows the typical dn-type elongation pattern of rice plant (Figs. 1C, 2D).

**Fig. 3.**
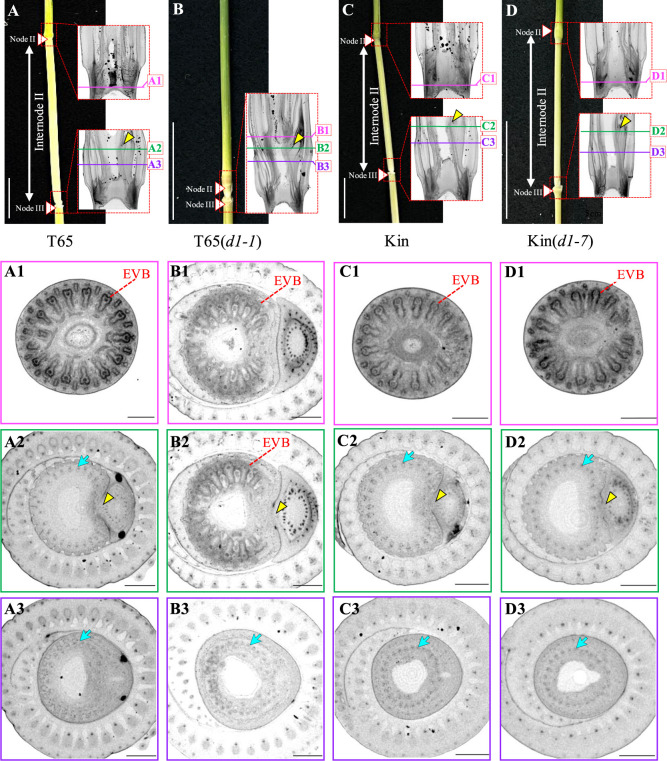
Anatomical observation of Internode II in the original cultivars and *d1* mutant lines. (A)–(D) Morphology of Internode II in T65 (A), T65(*d1-1*) (B), Kin (C) and Kin(*d1-7*) (D). While T65, Kin and Kin(*d1-7*) show a clear Internode II, T65(*d1-1*) does not. Framed areas indicate positions observed by micro-CT scanning. Horizontal lines with letters in longitudinal sections indicate the corresponding positions of transverse sections below. Bar, 5 cm. (A1)–(D1) Transverse sections of middle of Node II. (A2)–(D2) Transverse sections of the connecting point of Foot II domain with the upper domain. In a typical node–internode pattern, the upper domain of Foot II is Internode II. Yellow arrowhead, upper edge of Foot II where the axillary bud attaches the stem part. (A3)–(D3) Transverse sections of middle of Foot II. EVB, enlarge vascular bundle. Blue arrow, narrow vascular bundle. Bar, 1 mm.

Subsequently, we analyzed the internode elongation patterns of these mutant lines through anatomical observation of the Internode II. The rice shoot comprises successive phytomers, each consisting of a leaf, a stem, and an axillary bud. The stem part can be divided into three domains: an upper non-elongating domain (Node), an elongating domain (Internode), and a lower non-elongating domain (Foot) (Fig. 1B, Supplemental Fig. 1A). This arrangement represents a typical node–internode pattern in rice (Tanaka *et al.* 2023, Tsuda *et al.* 2023). Vascular bundles serve as clear landmarks to clarify this pattern within a phytomer. In the nodes, the vascular bundles are larger than those in the internodes and foots, and these node-specific vascular bundles, called enlarged vascular bundles (EVBs), are crucial for the preferential distribution of solutes (Yamaji and Ma 2014, 2017) (Fig. 3A1). In the internodes, the vascular bundles are narrow and longitudinally arranged, likely optimizing internode elongation (Fig. 3A2). In the foots, the vascular bundles are narrow as in the internodes but connect to those in the axillary bud (Tsuda *et al.* 2023) (Fig. 3A3). We investigated the structures of vascular bundles throughout the flag leaf phytomer, containing Node II and Internode II of the original cultivars and *d1* mutant lines, through micro-CT scanning (Supplemental Fig. 1A, Supplemental Video 1). In Fig. 3, we illustrate three representative positions: the middle of Node II (Supplemental Fig. 1A, Fig. 3A1–3D1), the upper edge of Foot II where the Foot II connects the upper domain (Supplemental Fig. 1A, Fig. 3A2–3D2), and the middle of Foot II (Supplemental Fig. 1A, Fig. 3A3–3D3). As reported (Tsuda *et al.* 2023), the original cultivars T65 and Kin exhibited EVBs in the middle of Node II (Fig. 3A1, 3C1). The mutant lines T65(*d1-1*) and Kin(*d1-7*) also had EVBs in the middle of Node II (Fig. 3B1, 3D1). At the upper edge of Foot II, where the Foot II connects to the Internode II, the original cultivars T65 and Kin, and the mutant line Kin(*d1-7*) had narrow vascular bundles (Fig. 3A2, 3C2, 3D2). Conversely, narrow vascular bundles were not observed at the upper edge of Foot II in the mutant line T65(*d1-1*), but it had EVBs (Fig. 3B2). The presence of EVBs from the Node II toward the upper edge of Foot II suggests that the Internode II seems to be lacked in the mutant line T65(*d1-1*). Additionally, in the foots, all four plants exhibited narrow vascular bundles, connecting to those in the axillary bud (Fig. 3A3–3D3). This reveals a connecting structure of Node II–Foot II–Node III, without Internode II, in stem of the mutant line T65(*d1-1*), resulting in a significantly short SIL (as distance from Node II to Node III) observed in this mutant (Fig. 2, Supplemental Fig. 1B). In contrast, the mutant line Kin(*d1-7*) produces a relatively elongated Internode II between the Node II and Foot II (Fig. 2, Supplemental Fig. 1B).

The *d1* mutant lines show abnormal phenotypes compared to the original cultivars. Both T65(*d1-1*) and Kin(*d1-7*) plants exhibit dwarf stature with erect dark-green leaves (Supplemental Fig. 2A, 2D), compact panicle type (Supplemental Fig. 2B, 2E), and small-round seeds (Supplemental Fig. 2C, 2F, 2G). These abnormal phenotypes were reported as pleiotropic effects of loss-of-function of the *DWARF1* (*D1*) gene, which encodes Gα-subunit of heterotrimeric GTP-binding proteins (Ashikari *et al.* 1999, Fujisawa *et al.* 1999). To confirm the mutation sites in the *d1* mutant lines compared to the original cultivars, we sequenced their *D1* cDNA. In T65(*d1-1*), there was a 2 bp-deletion at nucleotides 913-914 in the *D1* coding sequence (CDS). On the other hand, a 19 bp-insertion between nucleotides 264 and 265 in the *D1* CDS was present in Kin(*d1-7*). This data is consistent with the previous reports (Fujisawa *et al.* 1999, Oki *et al.* 2009a) (Supplemental Fig. 3A–3C). Due to different mutation sites in *D1* gene, we considered the possibility that the *d1* mutant alleles may cause differences in the elongation of Internode II between T65(*d1-1*) and Kin(*d1-7*). We proposed that the dm-type shortening of Internode II in T65(*d1-1*) is consistent with the severe disruption of *D1* protein function, impacting the signal transduction pathways necessary for internode development. The 19 bp-insertion is located at the junction of the 4^th^ exon and 4^th^ intron in the *D1* gene of Kin(*d1-7*) (Supplemental Fig. 3C), so we speculated that the *d1* allele in Kin(*d1-7*) might exhibit a leaky behavior due to mis-splicing. We hypothesized that Kin(*d1-7*) may express both the mutant and wild-type CDS of the *D1* gene. The expression of the wild-type *D1* CDS in Kin(*d1-7*) may contribute to the elongation of Internode II in this mutant line. To verify this, at first we checked the expression of the *D1* mRNA. The *D1* mRNA was similarly expressed in T65(*d1-1*) and Kin(*d1-7*) (Supplemental Fig. 3D). Subsequently, we cloned and sequenced *D1* cDNA of Kin(*d1-7*). All 17 clones yielded only the mutant type (19 bp-deletion) (Supplemental Fig. 3E). These results refute the possibility of the *d1* allele in Kin(*d1-7*) being a leaky allele, which would overall rescue the elongation of Internode II in Kin(*d1-7*). Additionally, T65(*d1-1*) and Kin(*d1-7*) were derived from different genetic backgrounds, T65 and Kin, respectively (Supplemental Fig. 2A). This variation raises further hypotheses: other loci, aside from *d1*, in different genetic backgrounds may influence determination of dm-type or dn-type patterns, or other loci with specific *d1* alleles may interact to determine the internode elongation patterns. Therefore, we initiated a genetic analysis to identify loci associated with the elongation of Internode II in these mutant lines.

### Detection of QTLs regulating the SIL of the *d1* mutant lines

We conducted reciprocal crosses between T65(*d1-1*) and Kin(*d1-7*) plants (Supplemental Fig. 4A). All nine F_1_ plants derived from the cross T65(*d1-1*)/Kin(*d1-7*) and five F_1_ plants derived from the cross Kin(*d1-7*)/T65(*d1-1*) displayed a dwarf phenotype like the parental lines T65(*d1-1*) and Kin(*d1-7*) (Supplemental Fig. 4B). Additionally, all these F_1_ plants exhibited an internode elongation pattern resembling that of the parental line Kin(*d1-7*), in which the length of Internode II was significantly longer than that of the parental line T65(*d1-1*) (Supplemental Fig. 4B, 4C). This suggests that the dominant gene(s) in Kin(*d1-7*) contribute to increased elongation of Internode II.

**Fig. 4.**
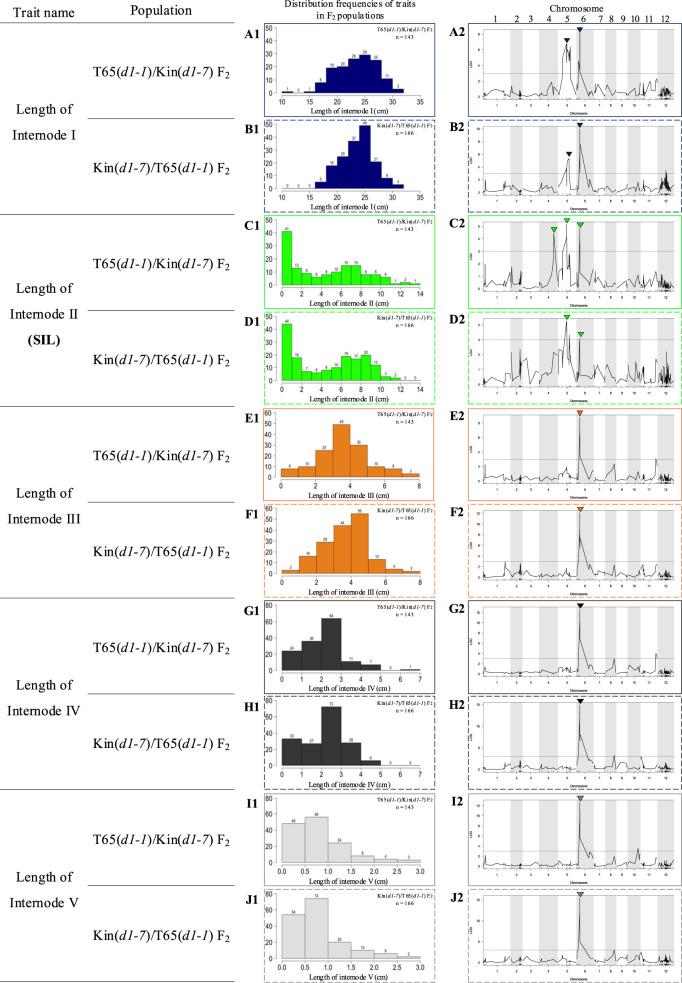
Detection of QTLs regulating the length of internodes of *d1* mutant lines. (A1–J1) Distribution frequencies of the traits: Length of Internode I (ocean blue, A1–B1), Length of Internode II or SIL (green, C1–D1), Length of Internode III (orange, E1–F1), Length of Internode IV (black, G1–H1), Length of Internode V (gray, I1–J1). (A2–L2) Detection of QTLs regulating the traits: Length of Internode I (A2–B2), Length of Internode II or SIL (C2–D2), Length of Internode III (E2–F2), Length of Internode IV (G2–H2), Length of Internode V (I2–J2). Major QTLs are marked by arrowheads, with detailed information provided in Table 1. Solid border represents T65(*d1-1*)/Kin(*d1-7*) F_2_ population and dashed border represents Kin(*d1-7*)/T65(*d1-1*) F_2_ population.

To identify the locus associated with the elongation of Internode II of the *d1* mutant lines, we conducted QTL analysis using the F_2_ generation originating from the F_1_ plants. We measured the length of Internode I–V in 143 F_2_ plants derived from the cross T65(*d1-1*)/Kin(*d1-7*) and in 166 F_2_ plants derived from the cross Kin(*d1-7*)/T65(*d1-1*) (Supplemental Fig. 4A). The internode lengths showed a wide distribution (Fig. 4A1–4J1). QTL analysis detected three significant QTLs for the SIL, designated as *qSIL4*, *qSIL5*, and *qSIL6*, on chromosomes 4, 5 and 6, respectively (Fig. 4C2, 4D2, Table 1). The QTL on chromosome 6 (*qSIL6*) was associated not only with the Internode II but also with other internodes (Fig. 4A2–4J2, Table 1). The QTL on chromosome 5 (*qSIL5*) also affects the length of Internode I (Fig. 4A2–4D2, Table 1).

**Fig. 5.**
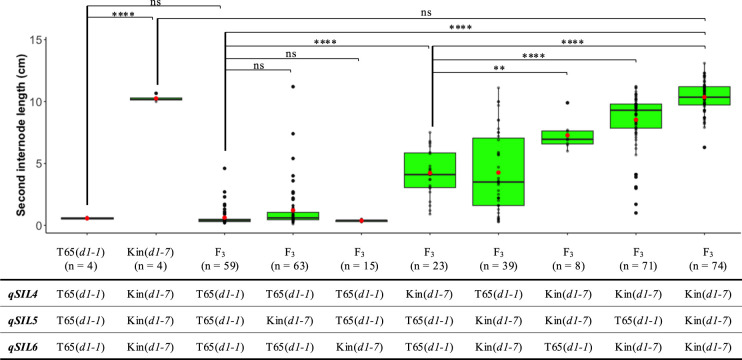
Effect of QTLs on the second internode length (SIL). F_3_ plants with homozygous genotypes of T65(*d1-1*) and Kin(*d1-7*) at *qSIL4*, *qSIL5* and *qSIL6* were grouped into eight combinations. The genotypes are based on markers at peaks of *qSIL4* (S04_24036340) and *qSIL6* (S06_8016433), and between flanking markers of *qSIL5* (from S05_14104696 to S05_17836992). In the box plot, red dot indicates mean, while horizontal bar indicates median of group. n, number of F_3_ plants in groups. Statistical analysis was performed using one-way ANOVA (α = 0.01), followed by Tukey’s multiple comparison test, * p < 0.05, ** p < 0.01, *** p < 0.001, **** p < 0.0001. ns, no significant difference.

To evaluate the effects and interaction of these QTLs, we selected 11 F_2_ plants segregating at the QTL regions to generate 11 F_3_ populations (F_3__01–F_3__11) (Supplemental Fig. 5A). We genotyped and examined internode phenotypes in a total of 760 F_3_ plants (Supplemental Fig. 5B). Only F_3_ plants with homozygous genotypes and no recombination between flanking markers at three QTL regions were selected and grouped into eight groups based on marker genotypes at *qSIL4*, *qSIL5* and *qSIL6* (Fig. 5). Significant interactions were observed among the three QTLs in the F_3_ populations (Supplemental Table 3, Fig. 5). Kin(*d1-7*) allele at *qSIL4* is sufficient for the elongation of SIL. In addition, combination of Kin(*d1-7*) alleles at *qSIL5* and *qSIL6* also caused the elongation of SIL.

## Discussion

The architecture of the plant stem, composed of alternating nodes and internodes, is critical for overall plant height. While nodes remain static, internodes possess the capacity for elongation. Key plant hormones such as GA, Auxin, and BR are known to enhance internode elongation. And in the last two decades, the molecular mechanisms, including biosynthesis and signal-transduction pathway, have been well-studied (Korasick *et al.* 2014, Kuroha *et al.* 2018, Nagai *et al.* 2020, Nolan *et al.* 2019). Despite these advances, the specific molecular factors behind the variation in internode elongation pattern remain unclear. This variability is particularly evident in rice, where distinct elongation patterns have been documented in dwarf mutants, as categorized by Takeda (1977) (Fig. 1C). These mutants serve as a valuable model for investigating genetic regulation of internode length. In this study, we examined the internode lengths and elongation patterns of two *d1* mutants of rice, namely T65(*d1-1*) and Kin(*d1-7*). Both mutants are characterized by loss-of-function mutations in the *D1* gene, yet they exhibit distinct internode elongation patterns: T65(*d1-1*) shows a dm-type pattern, with a specific shortening of Internode II, whereas Kin(*d1-7*) exhibits a dn-type pattern, characterized by uniform shortening across all internodes (Figs. 1C, 2).

To identify the genetic loci regulating the SIL, we performed a quantitative trait loci (QTL) analysis using two F_2_ populations derived from the reciprocal crosses of the *d1* mutant lines T65(*d1-1*) and Kin(*d1-7*). This analysis identified three significant QTLs, *qSIL4*, *qSIL5* and *qSIL6*, locate on chromosomes 4, 5, and 6 respectively, associated with the Internode II elongation. To assess the effects of these QTLs, we investigated the SIL of F_3_ plants carrying different genotype combinations at three QTLs. The F_3_ plants homozygous for T65(*d1-1*) alleles at all three QTLs showed a short SIL feature similar to the parental line T65(*d1-1*). Conversely, the F_3_ plants homozygous for Kin(*d1-7*) alleles at all these QTLs displayed a longer SIL feature resembling the parental line Kin(*d1-7*) (Fig. 5). These suggest that three Kin(*d1-7*) alleles collectively contribute to enhancing SIL. The candidate region of *qSIL5* includes many genes, but it contains the physical position of the *D1* gene. The T65(*d1-1*) and Kin(*d1-7*) plants display the loss-of-function phenotypes of the typical *d1* mutant (Supplemental Fig. 2). Moreover, the expression level of the *D1* did not significantly differ between T65(*d1-1*) and Kin(*d1-7*) (Supplemental Fig. 3D). The *D1* is pleotropic gene, which has multifunction for different traits (Ashikari *et al.* 1999, Fujisawa *et al.* 1999). Thus, if the *d1* mutant alleles are causative genes of the *qSIL5*, the mutant *d1* protein in Kin(*d1-7*) may still have function for the elongation of Internode II. This could be due to residual activity of the mutant protein or possibly due to other compensatory mechanisms in the plant. The *D1* gene in rice may exhibit spatiotemporal expression, which means its expression could vary both in time and space. This variation could be associated with the elongation of Internode II. It is important to note that the level of gene expression is not the only factor that determines a gene’s function. The timing and location of gene expression also play crucial roles in how the gene functions. For instance, Miura *et al.* (2009) reported on a specific mutant in rice, known as *Epi-d1*. This mutant is characterized by the ability to switch the *D1* gene expression on or off within the plant, demonstrating the potential for changes in gene expression to significantly impact the plant’s phenotype. Given these considerations, it is possible that the Kin(*d1-7*) may express the wild *D1* CDS from the elongation stage of Internode II and may partially rescue the elongation of Internode II.

We further characterized the dm-type and dn-type internode elongation patterns in the mutant lines T65(*d1-1*) and Kin(*d1-7*) by anatomical observations, focusing on the specific vascular bundle structures of nodes, internodes, and foots in rice (Tanaka *et al.* 2023, Tsuda *et al.* 2023). We examined the vascular bundle structures from Node II to Node III (assuming Internode II and Foot II segments within) of the mutant lines T65(*d1-1*) and Kin(*d1-7*) and their original cultivars using micro-CT scanning (Fig. 3, Supplemental Fig. 1). Our observations revealed that in Kin(*d1-7*) there is a sequential development from Node II, through Internode II, to Foot II. In contrast, T65(*d1-1*) produces Node II and Foot II, without the Internode II, leading to a significantly shortened SIL and resulting in the characteristic dm phenotype. These structural differences suggest variations in tissue differentiation and development. Recent findings of Tsuda *et al.* (2023) showed that cell fate determination in the rice stem begins just before leaf initiation at the flank of the shoot apical meristem (SAM), distinguishing cell lineages for leaves and stems. Cell fates for axillary buds are established early during leaf primordia development, with cells destined for internode development typically emerging from a few layers in the stem epidermis during the 12- to 25-cell stage. This progression implies that the development of internodes follows the determination of node cell fates. In T65(*d1-1*), the absence of initial cells that would typically develop into Internode II or disruptions in cell propagation and elongation processes likely contribute to the missing Internode II. A recent report by Tsuda *et al.* (2024) highlighted that abnormal expression of the *YABBY4* (*Os02g0643200*) and *YABBY5* (*Os04t0536300*) inhibits internode development in rice. Notably, the *d6* mutant, characterized by shortened Internodes II through V—except for Internode I—mis-expresses *YABBY4* and *YABBY5* in developing internodes, and loss-of-function mutations in these *YABBY* genes restored internode elongation in the *d6* mutant background. This indicates a significant regulatory role for *YABBY4* and *YABBY5* in internode formation, and *YABBY5* (26.8 Mb) coincides with *qSIL4* (23.6–29.9 Mb) on chromosome 4 (Table 1). There is a possibility that *YABBY5* might be the causative genes for *qSIL4* because T65(*d1-1*) allele at *qSIL4* shows more critical function for suppressing Internode II formation and/or elongation (Fig. 5), implying an overlapping function to *YABBY5*.

Furthermore, some BR-deficient rice mutants show reduced elongation specifically in Internode II (Hong *et al.* 2003, Niu *et al.* 2022, Oki *et al.* 2009b, Tanabe *et al.* 2005, Yamamuro *et al.* 2000). This suggests that BR plays a role in regulating Internode II in rice. The spatiotemporal production of BR in Internode II may be crucial for its normal elongation. The QTLs identified in this study could be involved in regulating the spatiotemporal production of BR. Cloning these QTLs and conducting functional analyses could provide further insights into their roles.

In conclusion, this study demonstrated the complex interaction of genetic factors influencing internode elongation in rice, with distinct patterns observed in two *d1* mutants. The identification of three significant QTLs provides a new understanding of the possible molecular mechanisms involved. This study represents an initial step toward addressing these gaps. Further research could potentially unlock new insights into plant growth and development, ultimately benefiting crop breeding strategies.

## Author Contribution Statement

Q.T.H and M.A designed the experiments. K.N and K.M generated the breeding materials. Q.T.H, S.M, V.P.R and K.D carried out the QTL analysis. Q.T.H, S.M and M.M confirmed the sequence and quantified the endogenous expression level of the *D1* gene. K.T and A.M performed the anatomical observation. Q.T.H and M.A wrote the manuscript.

## Supplementary Material

Supplemental Figures

Supplemental Tables

Supplemental Video

## Figures and Tables

**Table 1.** Detection of QTLs regulating the length of internodes of *d1* mutant lines*^a^*

Trait name	Population	Chr*^b^*	Marker at QTL peak*^c^*	QTL region (Mb)*^c^*	LOD*^d^*	AE*^e^*	PVE (%)*^f^*
Length of Internode I	T65(*d1-1*)/Kin(*d1-7*) F_2_	5	S05_13656667	7.4–19.6	6.8	1.6	19.7
		6	S06_8016433	8.0–8.1	8.2	2.0	23.3
	Kin(*d1-7*)/T65(*d1-1*) F_2_	5	S05_17416213	14.0–17.8	5.4	1.4	13.9
		6	S06_7982390	7.8–8.0	10.0	1.1	24.3
Length of Internode II (SIL)	T65(*d1-1*)/Kin(*d1-7*) F_2_	4	S04_24036340	23.6–29.9	4.4	1.7	13.3
		5	S05_13766373	7.4–14.0	5.2	1.7	15.3
		6	S06_7982390	5.7–8.1	4.9	1.9	14.5
	Kin(*d1-7*)/T65(*d1-1*) F_2_	5	S05_13656667	7.4–19.5	4.3	1.3	11.2
		6	S06_7839592	5.7–8.0	3.1	1.2	8.2
Length of Internode III	T65(*d1-1*)/Kin(*d1-7*) F_2_	6	S06_7982390	7.8–8.0	8.8	1.1	24.6
	Kin(*d1-7*)/T65(*d1-1*) F_2_	6	S06_7982390	7.8–8.0	12.1	0.9	28.5
Length of Internode IV	T65(*d1-1*)/Kin(*d1-7*) F_2_	6	S06_7982390	7.8–8.0	12.6	0.9	33.3
	Kin(*d1-7*)/T65(*d1-1*) F_2_	6	S06_7982390	7.8–8.0	14.9	0.8	33.8
Length of Internode V	T65(*d1-1*)/Kin(*d1-7*) F_2_	6	S06_7982390	7.8–8.0	12.6	0.5	33.4
	Kin(*d1-7*)/T65(*d1-1*) F_2_	6	S06_7982390	7.8–8.0	15.4	0.5	34.8

*^a^* QTL analysis data is shown in the Fig. 4. *^b^* Chromosome. *^c^* Physical localization of SNP marker at peak and QTL region based on Nipponbare IRGSP-1.0 reference genome (https://rapdb.dna.affrc.go.jp/index.html). *^d^* Logarithm of odds score. *^e^* Additive effect of the marker calculated as [mean of Kin(*d1-7*) – mean of T65(*d1-1*)]/2. Positive values indicate phenotypic direction to which the Kin(*d1-7*) allele increases the trait value. *^f^* Phenotypic variation explained by each QTL.
