# Supplementary material for: Detection of QTLs regulating the second internode length in rice dwarf mutant d1
Source: Breed Sci. 2024 Dec 3;74(5):443–53. doi: 10.1270/jsbbs.24036 (PMC11780330; doi:10.1270/jsbbs.24036)
Supplement: Supplementary file 1 — Supplemental Figures [file 74_443_s1.pdf]

Ha et al./Breeding Science
doi: 10.1270/jsbbs.24036

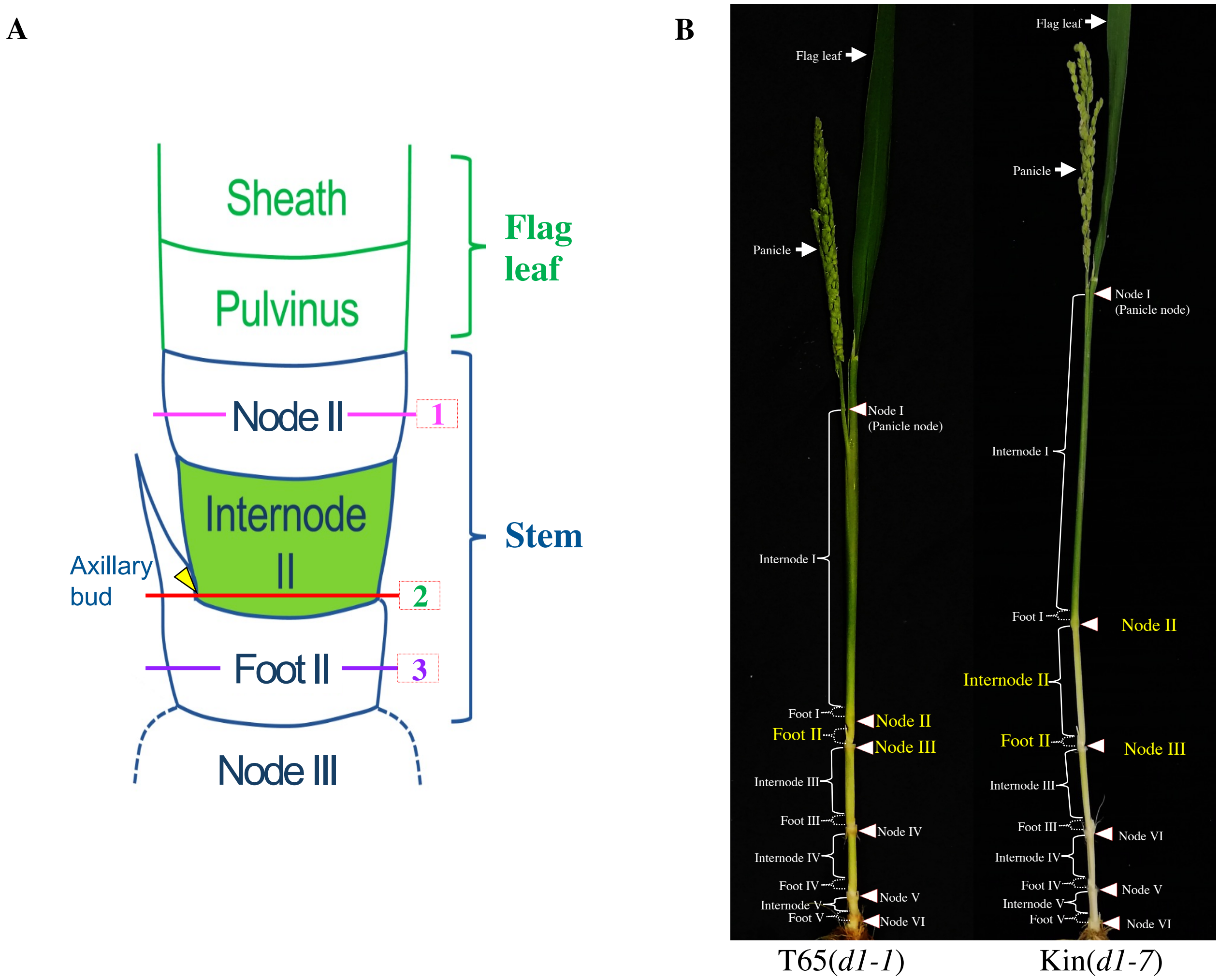

Supplemental Fig. 1. Node – internode pattern in *d1* mutant lines

(A) Illustration of domains in a typical node – internode pattern of the flag leaf phytomer, which was used for anatomical observation and described in Fig. 3, including the middle of Node II [1], the connecting point of the Foot II domain with the upper domain [2], and the middle Foot II [3]. Illustration was modified from Tsuda *et al*., 2023. **(B)** Node – internode pattern of *d1* mutant lines. Regions observed by micro-CT scanning are highlighted by yellow letters. Kin(*d1-7*) displays a normal node – internode pattern with Node II, Internode II, Foot II produced in order. On the other hand, T65(*d1-1*) lacks Internode II, exhibiting a connecting structure of Node II – Foot II – Node III.

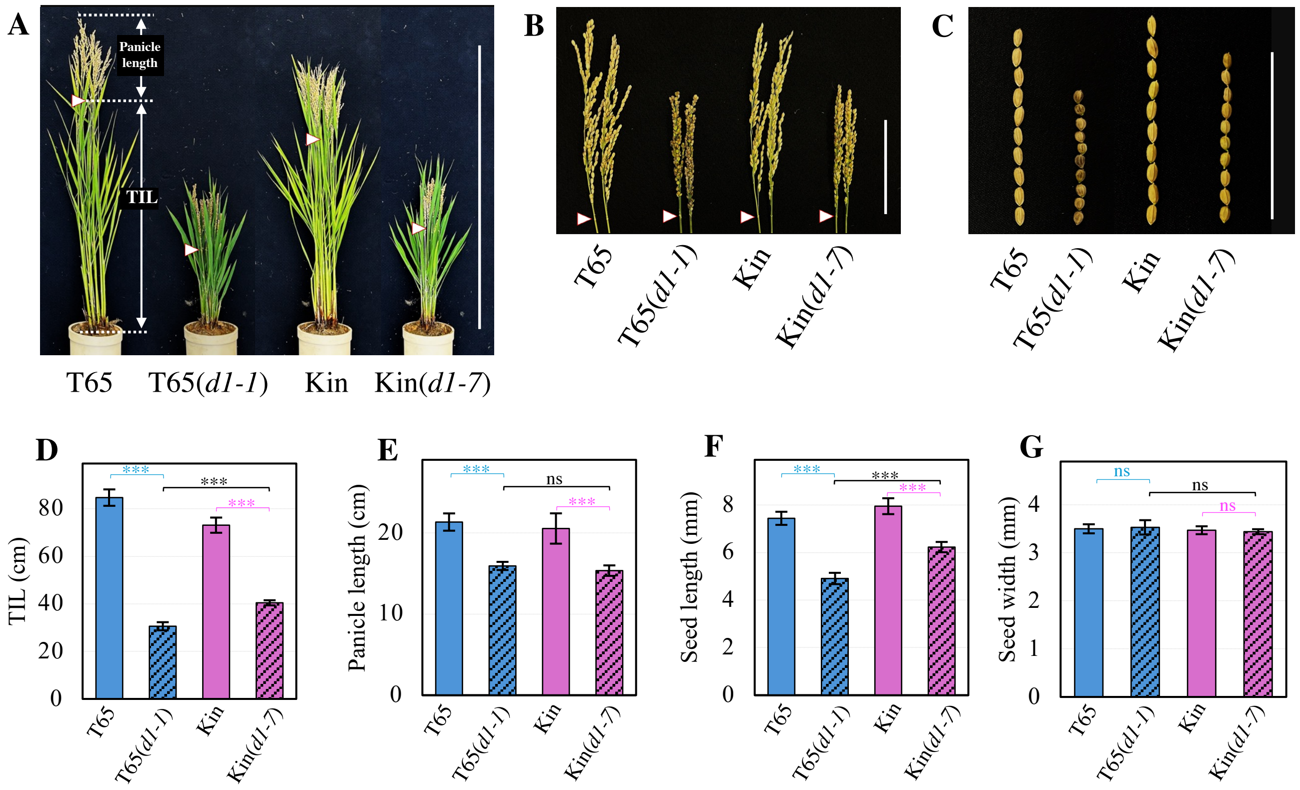

Supplemental Fig. 2. Phenotypic characterization of the original cultivars and *d1* mutant lines. **(A)** Gross morphology. The *d1* mutant lines show dwarf stature and erect dark-green leaves compared to the original cultivars. Arrowhead indicates Node I, which is the connecting point of the culm and the panicle. Bar, 1m. **(B)** Panicle morphology. The *d1* mutant lines show compact panicles compared to the original cultivars. Bar, 10cm. **(C)** Seed morphology. The *d1* mutant lines have small-round seeds compared to the original cultivars. Bar, 5cm. **(D) – (G)** Quantitative data of TIL (D), panicle length (E), seed length (F) and seed width (G). Values are means with SD, n = 6 in (D) and (E), n = 10 in (F) and (G). Statistical analysis by student's t-test, ***$p < 0.001$. ns, no significant difference. T65, cultivar 'Taichung65'. T65(*d1-1*), *d1* mutant line in background of T65. Kin, cultivar 'Kinmaze'. Kin(*d1-7*), *d1* mutant line in background of 'Kinmaze'.

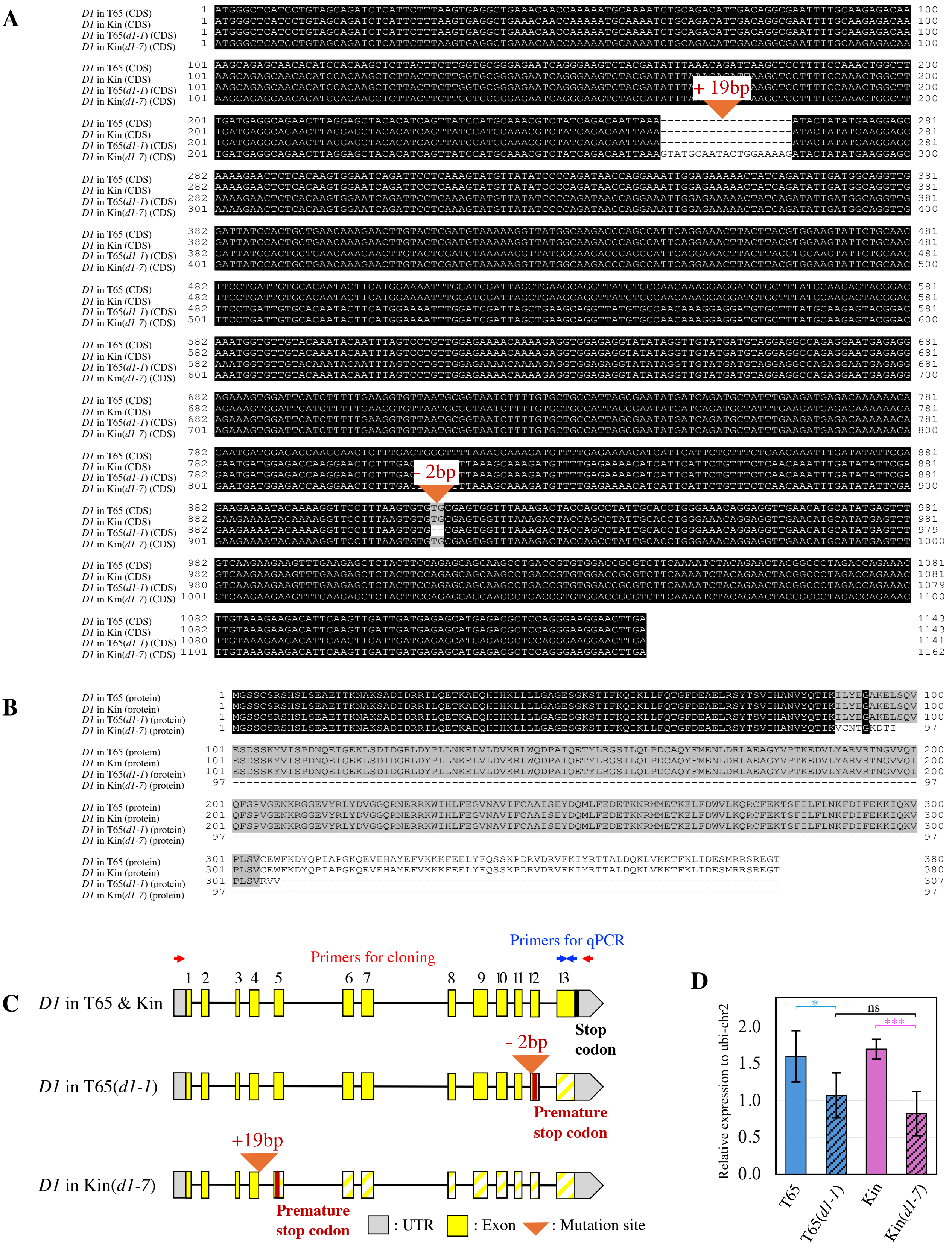

E

Clone	No. clones	Mutation
D1 in T65(*d1-1*)	10	2bp-deletion at nucleotides 913 - 914
D1 in Kin(*d1-7*)	17	19bp-insertion between nucleotides 264 and 265

Supplemental Fig. 3. *DWARF1* (*D1*) gene in the original cultivars and *d1* mutant lines.

Supplemental Fig. 3. *DWARF1* (*D1*) gene in the original cultivars and *d1* mutant lines. **(A)** Coding sequences (CDS) of *D1* proteins. **(B)** Amino acid sequences of *D1* proteins. The Genetyx software and CLUSTAL-W program were used for alignment in (A) and (B). **(C)** Structure of *D1* genes. **(D)** Relative expression level of *D1* genes by qPCR. Values are means with SD, n = 5. Statistical analysis by student's t-test, ***$p < 0.001$, *$p < 0.05$. ns, no significant difference. **(E)** The mutation sites detected in the clones of *D1* cDNA of *d1* mutant lines. The positions of primers used for qPCR and cloning *D1* cDNA of *d1* mutant lines are presented in (C).

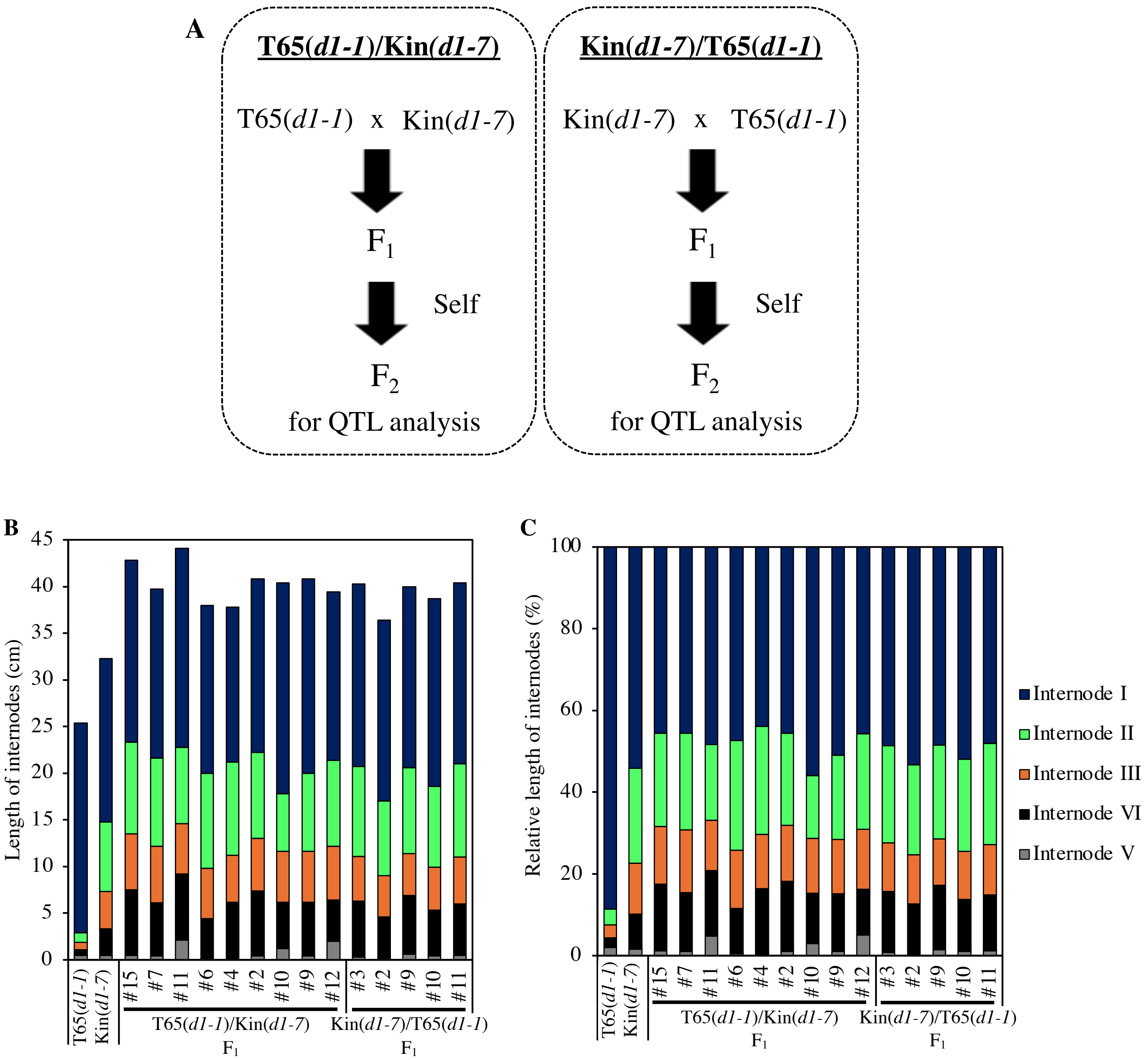

Supplemental Fig. 4. Internode elongation patterns of F_1 plants

(A) Breeding scheme of the breeding materials for QTL analysis. The mutant lines T65(*d1-1*) and Kin(*d1-7*) served as parental lines for reciprocal crosses. **(B)** Length of internodes of F_1 plants. **(C)** Relative length of internodes to the total internode length of the F_1 plants. All 14 F_1 plants show an internode elongation pattern look like that of the parental line Kin(*d1-7*).

A

Popu. name (No. plants)	Parental lines		Genotypes of F_2 original lines										
	T65(*d1-1*) (n = 4)	Kin(*d1-7*) (n = 4)	F_3_01 (n = 73)	F_3_02 (n = 64)	F_3_03 (n = 74)	F_3_04 (n = 64)	F_3_05 (n = 72)	F_3_06 (n = 72)	F_3_07 (n = 67)	F_3_08 (n = 71)	F_3_09 (n = 57)	F_3_10 (n = 67)	F_3_11 (n = 72)
qSIL4	T65(*d1-1*)	Kin(*d1-7*)	T65(*d1-1*)	T65(*d1-1*)	T65(*d1-1*)	T65(*d1-1*)	T65(*d1-1*)	hetero	hetero	hetero	hetero	Kin(*d1-7*)	Kin(*d1-7*)
qSIL5	T65(*d1-1*)	Kin(*d1-7*)	hetero	hetero	hetero	Kin(*d1-7*)	hetero	T65(*d1-1*)	hetero	hetero	hetero	T65(*d1-1*)	Kin(*d1-7*)
qSIL6	T65(*d1-1*)	Kin(*d1-7*)	T65(*d1-1*)	T65(*d1-1*)	hetero	hetero	Kin(*d1-7*)	T65(*d1-1*)	T65(*d1-1*)	hetero	hetero	Kin(*d1-7*)	Kin(*d1-7*)

B

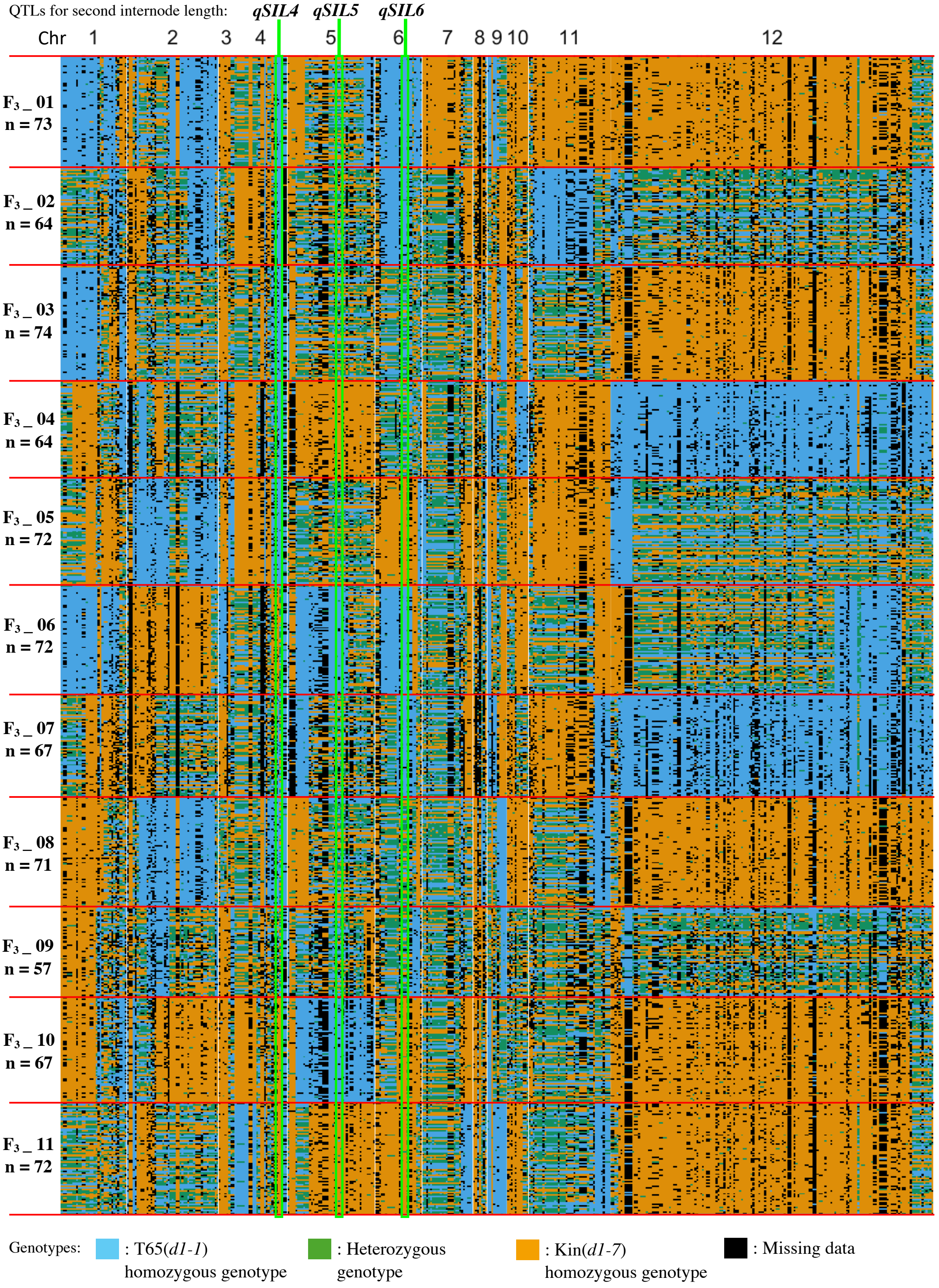

Supplemental Fig. 5. F_3 populations used for evaluation of QTL effect.

Supplemental Fig. 5. F_3 populations used for evaluation of QTL effect.

(A) Genotypes at *qSIL4*, *qSIL5* and *qSIL6* of F_2 original lines: T65(*d1-1*), Kin(*d1-7*) and hetero indicate T65(*d1-1*) homozygous genotype, Kin(*d1-7*) homozygous genotype and heterozygous genotype, respectively. F_3_01 ~ F_3_11, names of F_3 populations originating from the F_2 lines. n, number of F_3 plants in each population. **(B)** Genotypes of F_3 offsprings originating from F_2 lines. The genotypes are based on GBS genotyping data.
