# Supplementary material for: Detection of QTLs regulating the second internode length in rice dwarf mutant d1
Source: Breed Sci. 2024 Dec 3;74(5):443–53. doi: 10.1270/jsbbs.24036 (PMC11780330; doi:10.1270/jsbbs.24036)
Supplement: Supplementary file 2 — Supplemental Tables [file 74_443_s2.pdf]

Ha et al./Breeding Science
doi: 10.1270/jsbbs.24036

Supplemental Table 1. List of primers

	Name	Sequence	Physical location*
PCR amplification and cloning	56_d1-exon1-F	GATCCTGAGATCTAGACGTC	chr5: 15613587 - 15613568
	65_d1-exon13-R	ATCAATCAATGGTCCACGTG	chr5: 15609720 - 15609739
Sequencing	56_d1-exon1-F	GATCCTGAGATCTAGACGTC	chr5: 15613587 - 15613568
	671_d1-7_F	ACTTAGGAGCTACACATCAG	chr5: 15612843 - 15612824
	185_d1-RT-R1	GAGCGTCTCATGCTCTCATC	chr5: 15609860 - 15609879
	65_d1-exon13-R	ATCAATCAATGGTCCACGTG	chr5: 15609720 - 15609739
qPCR	184_d1-RT-F1	CTACAGAACTACGGCCCTAG	chr5: 15609934 - 15609915
	185_d1-RT-R1	GAGCGTCTCATGCTCTCATC	chr5: 15609860 - 15609879

* Physical localization is based on The Rice Annotation Project (https://rapdb.dna.affrc.go.jp/index.html)

Supplemental Table 2. Length of internodes of the *d1* mutant lines in 2021 – 2023

Year	Plant	n [a]	Panicle length [b]	Total internode length [c]	Internode I	Internode II	Internode III	Internode IV	Internode V
2021	**T65(*d1-1*)**	**10**	**16.3**	**27.1**	**23.9**	**0.6**	**2.4**	**0.7**	**0.0**
	**Kin(*d1-7*)**	**10**	**16.6**	**34.3**	**19.3**	**7.0**	**4.2**	**2.9**	**0.9**
	t-test [d] (p-value)		0.737	1.56×10^{-08}	5.67×10^{-09}	2.10×10^{-19}	1.23×10^{-04}	4.04×10^{-06}	1.31×10^{-05}
	significance level [e]		ns	***	***	***	***	***	***
2022	**T65(*d1-1*)**	**4**	**17.1**	**31.0**	**27.5**	**0.6**	**2.0**	**0.9**	**0.4**
	**Kin(*d1-7*)**	**4**	**16.0**	**43.9**	**23.5**	**10.3**	**5.4**	**3.4**	**1.2**
	t-test [d] (p-value)		0.146	4.99×10^{-05}	4.22×10^{-04}	9.29×10^{-10}	8.74×10^{-04}	2.14×10^{-05}	2.04×10^{-02}
	significance level [e]		ns	***	***	***	***	***	*
2023	**T65(*d1-1*)**	**6**	**15.9**	**30.6**	**25.4**	**0.4**	**3.7**	**0.9**	**0.3**
	**Kin(*d1-7*)**	**6**	**15.4**	**40.4**	**21.4**	**9.6**	**4.7**	**3.2**	**1.2**
	t-test [d] (p-value)		0.114	3.00×10^{-07}	1.39×10^{-05}	5.27×10^{-13}	9.67×10^{-03}	4.44×10^{-07}	1.63×10^{-05}
	significance level [e]		ns	***	***	***	**	***	***

[a] Number of plants used for phenotyping

[b] The distance from the node I to the tip of panicle

[c] The total length of measured internodes.

[d-e] Statistical analysis by student's t-test, ***$p < 0.001$, **$p < 0.01$, *$p < 0.05$. ns, no significant difference.

Supplemental Table 3. ANOVA among the QTLs for SIL in F_3 populations

Effect and interaction of QTLs	df	Mean square	F-value	Probability (p-value) [a]	Significant level [b]
qSIL4	1	4385	1281.419	0.0000	***
qSIL5	1	419	122.452	0.0000	***
qSIL6	1	460	134.413	0.0000	***
qSIL4 x *qSIL5*	1	16	4.809	0.0290	*
qSIL4 x *qSIL6*	1	58	16.880	0.0000	***
qSIL5 x *qSIL6*	1	31	9.064	0.0028	**
qSIL4 x *qSIL5* x *qSIL6*	1	63	18.519	0.0000	***

[a-b] Statistical analysis by ANOVA, ***$p < 0.001$, **$p < 0.01$, *$p < 0.05$.
